# Experiences with household mold and perceptions of microbiome engineering to mitigate mold

**DOI:** 10.3389/fpubh.2026.1725172

**Published:** 2026-01-27

**Authors:** Nourou Barry, Kristen D. Landreville, Denene Blackwood, Jada K. Yard, Rachel Noble, Jennifer Kuzma

**Affiliations:** 1 Genetic Engineering and Society Center, North Carolina State University, Raleigh, NC, United States; 2 Department of Communication and Journalism, University of Wyoming, Laramie, WY, United States; 3 Institute of Marine Sciences, University of North Carolina at Chapel Hill, Morehead City, NC, United States; 4 University of North Carolina at Chapel Hill, Chapel Hill, NC, United States; 5 School of Public and International Affairs, North Carolina State University, Raleigh, NC, United States

**Keywords:** built environment (BE), health belief model, household molds, microbiome engineering, mold prevention, mold remediation, semistructured interviews

## Abstract

**Background:**

Mold, biologically defined as fungal mold, is frequently identified as a household concern, especially in humid and coastal regions where conditions favor growth. Yet, the ways residents recognize, experience, and manage mold in their daily lives remain understudied. In recent years, emerging mold-control strategies, such as microbiome-engineering technologies, are increasing in application and attracting the interest of homeowners. However, these technologies raise important questions about community perceptions, trust, and acceptance. Understanding how people navigate everyday mold management and views on novel interventions is essential for guiding effective, market-relevant, socially-responsive solutions.

**Methods:**

This qualitative study draws on 22 interviews with residents of eastern North Carolina, an area with climate conditions that favor fungal molds. Using the Health Belief Model (HBM) as a framework, we explored participants’ conceptualizations of mold, perceived health and structural impacts, prevention strategies, and views on microbiome-engineering remediation technologies.

**Results:**

Residents understood mold in diverse ways, as a fungus, a dampness-driven growth, and a sensory presence tied to smell and sight. Mold was linked to respiratory illness, systemic health effects, property damage, and financial burdens. Participants employed layered strategies such as ventilation, dehumidification, cleaning, and occasional professional remediation, though cost, trust, and perceived effectiveness of these strategies remained barriers. Analysis through the HBM revealed high perceived severity of the mold problem and related health illness but underestimation of susceptibility due to reliance on visible cues. Reactions to microbiome-engineered tools showed cautious interest: while residents acknowledged potential benefits, they expressed concerns about unintended consequences, invisibility, and loss of control. Conditional acceptance was contingent on rigorous testing, transparent regulation, and proven safety and efficacy.

**Conclusion:**

Mold is experienced by Eastern NC residents not only as a biological contaminant but as a lived, socio-environmental challenge shaped by health, housing, and financial vulnerabilities. Participants in this study indicated serious health concerns related to mold, including after specific events such as large storms and flooding. Acceptance of microbiome-engineering solutions will depend on building trust, addressing equity, and ensuring accessibility. By bridging environmental science, social science, and residents’ lived experiences, policies and technologies can more effectively strengthen resilience against one of the most persistent risks in the built environment.

## 1 Introduction

Mold growth in residential environments is a persistent and common issue that many homeowners encounter, with far-reaching implications for household well-being, environmental quality, and social equity. Although mold spores are present in virtually all homes, it is only under certain conditions, such as elevated humidity, persistent dampness, and warm temperatures, that mold colonization becomes a significant problem (1–4). In the United States, homes located in the Southeast, Gulf Coast, and Pacific Northwest are particularly vulnerable due to their warm, humid climates and frequent rainfall, often resulting in chronic mold issues that can undermine both physical health and overall well-being (5–8). Moreover, older housing stock and inadequate building maintenance in many US communities contribute to widespread dampness and mold growth, exacerbating health risks for millions of Americans and other populations worldwide (9–12). Growing policy attention, such as US Representative Haley M. Stevens’s H. R.2746 Fix Moldy Housing Act [introduced April 8 (13)], underscores the increasing concern about indoor mold exposure (13).

The health consequences of indoor mold exposure are well documented, ranging from mild allergic reactions to severe respiratory illnesses, including asthma exacerbation and chronic sinusitis (8, 14–19). According to the Centers for Disease Control and Prevention (CDC), approximately 21 million adults in the US have asthma, with indoor mold exposure serving as a significant trigger (20). Beyond physical health, mold infestations can also contribute to psychological distress, stigma, and diminished quality of life, particularly among vulnerable populations (21–24).

Despite these well-established risks, the effectiveness of public health campaigns and remediation strategies remains uncertain. Federal agencies such as the Environmental Protection Agency (EPA) and state health departments provide guidance on moisture control and mold cleanup, yet there is limited evidence regarding the impact of these interventions on actual household mold levels or on the behaviors and attitudes of residents (5, 25–27). There are also no minimal standards (or threshold limit values) of mold exposure established for indoor environments based on dose–response relationships, and quantitative microbial risk assessments (QMRA) for general mold exposure are hindered by the lack of data linking specific exposures to predictable health outcomes (28, 29). Most research to date has focused narrowly on the biomedical effects of mold exposure, often neglecting the complex interplay between individual knowledge, lived experience, perceived risk, and remediation practices (1, 15, 30–34). As a result, critical questions remain about how people interpret mold risks, what motivates them to take action, and how they respond to both conventional and emerging, microbiome-engineering remediation strategies.

To address these gaps, this study draws on 22 in-depth interviews with homeowners residing in Carteret County, North Carolina, a rural coastal region characterized by its humid climate and recurrent storm-related water intrusion that creates conditions for household mold (35, 36). Climate projections suggest increasing storm intensity and continued coastal development, further elevating the relevance of mold-related investigations. The interviews approach deployed here covered topics such as how participants defined mold, their reactions to mold across an array of homeowner concerns, and perceptions of microbiome engineering to mitigate mold. For example, genetically engineered microbes could be deployed to displace pathogenic mold or engineered mycoviruses could be used to kill disease-causing fungi. The Health Belief Model (HBM) (37, 38), outlined below in the background section, served as the conceptual foundation for this study. It offers a framework to examine risk perception and health behaviors in relation to visible cues, personal experiences, and cultural narratives. The HBM is also well suited for exploring public attitudes toward innovative remediation technologies, such as the introduction of genetically engineered microbiomes (39, 40), which are increasingly being considered as proactive and adaptive solutions for mold management. The findings from this study aim to inform the development of more effective public health campaigns, targeted educational interventions, and evidence-based policies, while also supporting the responsible introduction of novel remediation technologies that align with the needs, values, and concerns of affected communities.

### 1.1 Background

Mold spores generated by fungal molds are a constant presence in indoor environments, but problematic growth is typically triggered by persistent moisture, inadequate ventilation, and warm temperatures (1–4). Geographic and structural factors further compound this issue: homes in humid regions such as the Southeast, Gulf Coast, and Pacific Northwest, as well as older or poorly maintained buildings, are especially susceptible to chronic mold problems (5–12). The adverse health effects of mold exposure are well established, with links to allergic reactions, respiratory symptoms, and exacerbation of chronic conditions like asthma (8, 14–19). These impacts are not limited to physical health; psychological distress and social stigma are also common, particularly among those in vulnerable or low-income communities (21–24).

Recent advances in environmental science have improved mold detection and identification, with technologies such as DNA-based sequencing, MALDI-TOF mass spectrometry, and real-time chemical sensors for mold metabolites offering more precise assessments (41, 42). However, these methods face limitations, including variability in indoor fungal ecology, lack of standardized health thresholds, and the need for comprehensive reference databases (41, 42). As a result, remediation decisions remain complex and context dependent. Innovative approaches, such as the introduction of genetically- engineered microbiomes, are emerging as promising tools for both identifying and neutralizing harmful fungi (39, 40). Yet, public acceptance of these technologies is shaped by perceived benefits, ethical considerations, and trust in regulatory oversight. Understanding these attitudes is crucial for the successful adoption of new interventions (39, 40). The literature increasingly calls for integrated strategies that combine technological innovation, behavioral science, and policy reform. Effective mold management requires not only advanced detection and remediation tools but also education, community engagement, and structural interventions that address the root causes of dampness and housing inequality (15, 22). As climate change intensifies moisture risks, adaptive and interdisciplinary responses will be essential (3, 43).

Given these complexities, the Health Belief Model (HBM) offers a relevant framework for this study, enabling a nuanced analysis of how individuals perceive mold risks, what motivates their responses, and how they evaluate both conventional and novel remediation strategies.

#### 1.1.1 The health belief model, mold risk perceptions, and attitudes toward genetically engineered microbiomes

The Health Belief Model (HBM) offers a robust theoretical framework for interpreting how individuals perceive, experience, and respond to mold in their homes, as well as their openness to novel remediation strategies such as genetically engineered microbiomes. Originating in the 1950s, the HBM was developed to explain why people often fail to engage in preventive health behaviors, even when the benefits are clear and accessible (37, 38). At its core, the HBM posits that health-related actions are shaped by perceived susceptibility to a health threat, perceived severity of its consequences, perceived benefits of taking action, and perceived barriers to action. Later expansions of the model introduced constructs such as cues to action and self-efficacy, further enriching its explanatory power (37).

Applying the HBM to the context of household mold reveals several critical insights. First, perceived susceptibility and severity are often shaped by both visible and invisible cues. Studies have shown that many residents underestimate their risk if they rely solely on visible mold, overlooking hidden or airborne contamination (14, 44). This perceptual gap can lead to a false sense of security and reduced motivation for remediation. Conversely, heightened awareness, whether triggered by media reports, health symptoms, or advanced diagnostic tools, can increase perceived risk and prompt action (30, 45). The HBM also illuminates the role of perceived benefits and barriers in shaping attitudes toward innovative solutions, such as genetically engineered microbiomes for mold remediation. While the promise of targeted, sustainable interventions may appeal to those who recognize the limitations of conventional cleaning or chemical treatments, skepticism about safety, efficacy, or unintended consequences can serve as significant barriers (15, 41). Here, cues to action, such as public health campaigns, endorsements from trusted authorities, or personal experiences with persistent mold, can be pivotal in shifting attitudes and behaviors.

Importantly, the HBM emphasizes that interventions must consider both individual understanding and environmental factors. For example, new tools for mold detection (such as qPCR testing and ERMI scores) can make risks from mold exposure clearer and easier to act on. This helps people feel more confident in taking preventive or corrective steps and reduces obstacles that might otherwise keep them from addressing the problem (46). However, these tools must be accompanied by clear communication and support, particularly for populations facing socioeconomic or informational barriers (22, 23). Overall, the HBM provides a valuable structure for understanding individual decision-making, and it offers a nuanced framework for understanding how people interpret and respond to mold risks and how they might evaluate emerging solutions like genetically engineered microbiomes. By attending to the interplay of perceived risk, benefits, barriers, and cues to action, this manuscript contributes to designing more effective interventions that resonate with lived experience and addresses both psychological and practical dimensions of mold management.

#### 1.1.2 An overview of mold literature

Over the past two decades, mold research has expanded, reflecting advances in science, building technology, and public health. An example of that advancement can be seen in the inclusion of mold and moisture criteria in the International Well Building Institute WELL Building Standard (47). Early studies focused on biological and material aspects, documenting prevalence, health impacts, and conditions promoting growth (17, 45). More recent work includes behavioral science, risk perception, and technological innovations for a broader understanding (12, 15).

One of the central themes from this literature is the inadequacy of relying solely on visible indicators or material assessments to gage mold risk. Studies have demonstrated that visible mold or moisture problems are poor predictors of airborne mold concentrations, challenging the assumption that only visibly affected homes pose health risks (44, 48). Most of the time, mold contamination is widespread, with concentrations shaped by both occupant behaviors (e.g., ventilation and cleaning habits) and building characteristics. Without comprehensive assessment, many residents may underestimate their exposure, leading to delayed remediation (49). Also, modern building practices, such as airtight construction and high insulation, have been shown to increase susceptibility to hidden mold growth, particularly when ventilation is inadequate (50, 51).

The health implications of mold exposure are well established. In the United States, mold and poor indoor air quality are consistently linked to respiratory infections, bronchitis, asthma, and mental distress (17, 22, 32, 52). Children in homes with significant moisture damage are more likely to experience persistent wheezing, and high mold levels are a risk factor for asthma (16, 30). Meta-analyses reinforce the association between indoor dampness, mold, and the development of new asthma cases (17, 32, 52). The literature also highlights the psychosocial burden of mold exposure, with increased anxiety, stress, and depression reported among residents of cold, damp, and mold-infested homes (22). Public perceptions in the US reflect these concerns. For example, in rural Alaska, mold and moisture were the most common indoor air quality issues for about 27% of participants, and addressing mold was seen as key to improving air quality (53). Despite this alignment between public concern and scientific evidence, interventions to improve health in mold-infested areas are infrequently discussed, and practical solutions remain rare in public health discourse.

Methodological advances have enriched the field, with microbiological analyses of building materials revealing a wide range of mold species and secondary metabolites (54), and air sampling techniques such as the Andersen sampler and quantification of N-acetylhecosaminidase (NAHA) activity providing more nuanced assessments (44). However, many advanced detection methods require laboratory analysis or specialized equipment, limiting their immediate applicability for routine monitoring or rapid remediation (54). Translating objective measurements into actionable guidance for occupants and developing universally accepted protocols for risk assessment remain ongoing challenges (12). Standardized metrics such as the Environmental Relative Moldiness Index (ERMI) have enabled more precise quantification of mold contamination and its health impacts (46, 55), but their applicability across different climates and building types is still limited, and their use in isolation may overlook the broader context of occupant behavior and risk perception.

The literature increasingly emphasizes the integration of behavioral and technological interventions for effective mold management. Strategies such as source control, improved ventilation, and air purification are recognized as essential, while the limitations of current remediation technologies and the need for ongoing public education are acknowledged (51, 56). The persistent gaps between scientific knowledge, public perception, and actionable solutions highlight the need for interdisciplinary collaboration to develop innovative technologies that mitigate the risks associated with mold exposure and enhance health outcomes for affected populations.

#### 1.1.3 Research on genetically engineered microbiomes in the built environment

The microbiome engineering field has rapidly advanced, with engineered microbiomes for the built environment being viewed as a transformative tool for enhancing indoor air quality and reducing contaminants, such as mold. Currently, only a limited amount of scholarship has been published on this topic, including those by Cummings (39, 40), Hardwick et al. (57), McBee et al. (58), and Singh and Rastogi (59). These works emphasize both the technical capabilities and the complex sociotechnical factors affecting the development and potential implementation of these innovations. At the core of this emerging paradigm is the notion that indoor microbial communities can be intentionally designed using CRISPR-edited consortia, engineered biocomposite materials, or precision inoculation strategies to suppress pathogenic fungi, degrade pollutants, and foster healthier indoor ecologies (40, 59). These interventions are not limited to passive environmental modification; rather, they represent a shift toward active, responsive, and even regenerative systems that can sense, adapt, and self-repair in response to environmental cues (58).

The translation of these technologies from laboratory to lived environment is far from straightforward. As Cummings et al. (40) demonstrate through nationally representative surveys, public attitudes toward genetically engineered (GE) microbiomes are shaped by a constellation of factors, including age, trust in science, perceived knowledge, and emotional responses to microbes. While younger, science-trusting individuals are more open to these innovations, support is highly conditional—hinging on demonstrable health benefits, controllable risks, and robust regulatory oversight. Notably, the desire for technological fixes coexists with entrenched beliefs about personal responsibility and cleanliness, creating a behavioral ambivalence that may complicate adoption and maintenance of engineered systems. Moreover, negative emotions such as disgust or fear can amplify perceived risks, particularly when interventions are described in ways that evoke strong affective responses (39). These findings underscore the importance of integrating public values, inclusivity, and transparency into the research and innovation process.

The ethical and societal dimensions of microbiome engineering are further complicated by issues of autonomy, consent, and justice. Hardwick et al. (57) warn that indoor microbiome interventions can inadvertently change occupants’ microbiota without their knowledge or consent, raising concerns about personal control. Social inequalities affect who bears risks and benefits: low-income and minoritized groups face more mold and poor air quality but less access to remediation and green spaces, risking increased health disparities if not managed equitably. While ethical frameworks exist, covering beneficence, non-maleficence, autonomy, and justice, there is a lack of empirical research on how these principles are applied and perceived in real-world settings, particularly among marginalized groups (57).

From a technical perspective, the development of living, regenerative materials, such as the fungal–bacterial biocomposites described by McBee et al. (58), represents a paradigm shift in how we conceptualize and interact with the built environment. These materials challenge the traditional dichotomy of “good” versus “bad” microbes, instead positioning certain fungi and bacteria as partners in sustainable construction, environmental monitoring, and even self-healing architecture. Yet, the successful integration of such materials into everyday spaces will depend not only on their technical performance but also on public perceptions of safety, controllability, and the visible presence of living organisms.

## 2 Method

This study used a qualitative approach with semi-structured interviews to explore residents’ perceptions of mold, their experiences, health and social impacts, and attitudes toward microbiome engineering interventions. Below, we describe the study, participant recruitment, interview method, and data analysis approach.

### 2.1 Study site

To answer the study’s research questions, 22 participants in the coastal North Carolina region were recruited for a home observation and interview. Coastal North Carolina is a relevant study area for household mold because it has high relative humidity (about 75–80%; (36)), a warm annual average temperature (about 63 degrees Fahrenheit; (60)), a high probability of precipitation that ranges from about 25 to 50% on any given day (61), a severe risk from flood (62, 63), and a substantial vulnerability to storms and hurricanes (69), which are all conditions that increase household mold vulnerability (35). Climate change will likely exacerbate these conditions. Indoor mold problems have been reported in coastal NC communities, such as in public schools after Hurricane Florence in 2018 (64).

Thus, homeowners in six towns located in coastal Carteret County, NC, were recruited. As of July 2024, Carteret County had just more than 70,000 residents, with 27.9% of the population 65 years or older, 51.0% female, and 86.4% of the population identified as white alone and not Hispanic or Latino (65). The median household income was just more than $70,000 and 32.3% of residents earned a bachelor’s degree or higher. We focused on homeowners because they are typically responsible for mold remediation decisions, have direct financial exposure to property damage, and are a key target population for microbiome-engineering interventions. As such, transferability is most appropriate to similar coastal, homeowner populations; experiences of renters and other housing situations may differ and warrant future study.

### 2.2 Participants

The participants were recruited through digital flyers shared to local social media and email groups and supplemented with word of mouth in the community and snowball sampling (where participants connected us with additional participants). Participants were recruited across the county, with 8 from Beaufort, 7 from Morehead City, 2 from Emerald Isle, 2 from Newport, 2 from Stella, and 1 from Cape Carteret. Interviews and home observations occurred between August 2024 and September 2025. No participants withdrew from the study after they were recruited and interviewed.

The median age of participants was 48, with a range of 27 to 83. There were 9 men and 13 women. Highest educational attainment varied, with 11 completing an advanced college degree, 8 completing a bachelor’s degree, 1 completing an associate’s degree, and 2 completing a high school degree. Income levels varied as well: 8 participants reported an annual household income below $100,000; 9 participants were between $100,000 and $150,000; and 5 participants earned more than $150,000. There were 15 participants who were employed and 7 participants who were retired. One participant identified as black, 20 participants reported being white, and one identified as a white Hispanic. There were 17 participants who were married or living with a long-term partner, 3 participants who were single, and 2 who were widowed. There were 9 households with children under age 18 living in the home.

### 2.3 Interviews

The interview questions were developed by a transdisciplinary team of scholars with backgrounds in social science, communication, biotechnology, and microbiology. The interviews were recorded using an audio recording device and were transcribed by a professional transcription company. The average interview time was 40 min, with interviews ranging from 26 min to 54 min.

After arrival at the participants’ home, the researchers verbally reviewed the informed consent and asked participants to review and sign the informed consent before the interview and home observation began (the home observation entailed documenting signs of visual mold; however, the results of the home observation are not reported in this current publication). The semi-structured interview began with 11 brief demographic questions (e.g., age, income, education, gender, etc.) that took just a few moments to answer. Then, participants were asked one question each about conceptualizations of mold, dangers of mold, health impact perceptions of mold, general household experiences with mold, observations of mold growth patterns, reactions to mold, and remediation awareness and concerns.

Next, a 142-word description of microbiome engineering was read to the participant. The paragraph defined key terms (e.g., “microbe,” “built environment”), explained the purpose microbiome engineering (i.e., to create beneficial, healthier indoor environments), given an example of the technology (e.g., creating mold-detecting HVAC systems), and acknowledged the existence of unintended negative consequences. After the description was read, the interviewer asked six questions to the participants that addressed their questions, feelings, concerns/risks, perceived benefits, willingness to adopt the technology, and preference for naturally occurring microbes versus genetically engineered microbes.

For the last set of questions, participants were asked about their mold observations in their community (e.g., if they had seen mold growing in particular locations or built environments), ideas for addressing mold (i.e., how can the community overcome mold problems), comparison of mold to other priorities, and final thoughts. See Supplementary material for the full interview protocol.

### 2.4 Data analysis

The interview data was analyzed using NVivo 14 software (66). We employed a combination of deductive and inductive coding methods. Deductive codes were derived from the interview discussion guide (e.g., mold experiences, prevention strategies, perceptions of microbiome-engineering technologies), while inductive codes emerged through line-by-line open coding of transcripts, allowing unanticipated themes to surface. Although the HBM was not used *a priori* for deductive coding, HBM concepts emerged inductively and subsequently guided our interpretation. Codes were operationalized through iterative refinement, with definitions and boundaries clarified as coding progressed.

The first author conducted coding in NVivo, with regular team discussions to review emerging codes, interpret patterns, and ensure analytical rigor. The research team engaged in discussions to interpret and assess the suitability of the codes. Thematic saturation was monitored iteratively and reached at approximately 15 interviews. Saturation was more robust among women (the majority of participants) than men, which we acknowledge as a limitation for transferability to male homeowners.

We deployed thematic content analysis to pinpoint key topics and patterns (perception of mold, dangers of mold, mold prevention and control strategies, perceptions of microbiome engineering as potential solutions, etc.) within the interview transcripts, enabling the systematic extraction of recurrent themes present in the data. NVivo facilitated: (1) organization and retrieval of coded segments for each theme, (2) matrix coding queries to examine patterns across demographic subgroups (e.g., gender, income, race/ethnicity, flood history), and (3) organization and visualization of code relationships to support theme development and refinement.

## 3 Results

### 3.1 Participants’ conceptualization of mold

Participants described mold using four main ideas: its biological classification, the conditions that facilitate its growth, its sensory characteristics, and its communal aspects. The following table summarizes how participants define and describe mold in their own words (Table 1).

**Table 1:** Examples of participants’ conceptualizations of mold.

Idea	Characteristic	Illustrative quotes
Biological nature	Fungus/Spore-Producing	“a fungus, microscopic that turns stuff all fuzzy” (**P7**); “It’s a fungus that produces spores that transport in the house.” (**P13**); “Well molds are fungus. They thrive on moisture, and they are transmitted by spores.” (**P16**).
Environmental factors	Moisture/Dampness	“It’s what grows when it’s moist” (**P3**); “It grows in damp environments, very dark. It does not like the sunlight.” (**P12**); “They love moisture, and they’ll find a moist spot to grow.” (**P13**).
Physical appearance	Color/Texture/Visibility	“It’s just generally a little spotty and then it gets a little thicker looking and it fills itself in. I suppose it gets fuzzy sometimes too.” (**P15**); “A certain look, like small, faint, black spots on the drywall or on the fiberglass tub/shower enclosure.” (**P17**); “Green black mold; there are different colors of mold. Furry. Definitely.” (**P19**).
Sensory cues	Smell	“fuzzy appearance and unpleasant odor” (**P7**); “Just the smell, like you can smell it before you see it, right, that mildewy, musty.” (**P12**); “I can start smelling it. So, I feel like I would know the scent of it I suppose. Damp, mildewy; that kind of smell.” (**P19**).

The findings reveal that participants define mold as biological, specifically a fungus, which reproduces via spores and relies on moisture and dampness for growth. Furthermore, participants describe mold through its observable characteristics, noting variations in color (such as green, black, or orangey-gray) and texture (spotty, thick, fuzzy). Equally important are the sensory cues, particularly the distinctive musty or mildewy smell. Together, these elements form an experience-based definition of mold in their daily lives.

### 3.2 Participants’ perceptions of mold danger and impact

#### 3.2.1 Respiratory issues

**Figure 1:**
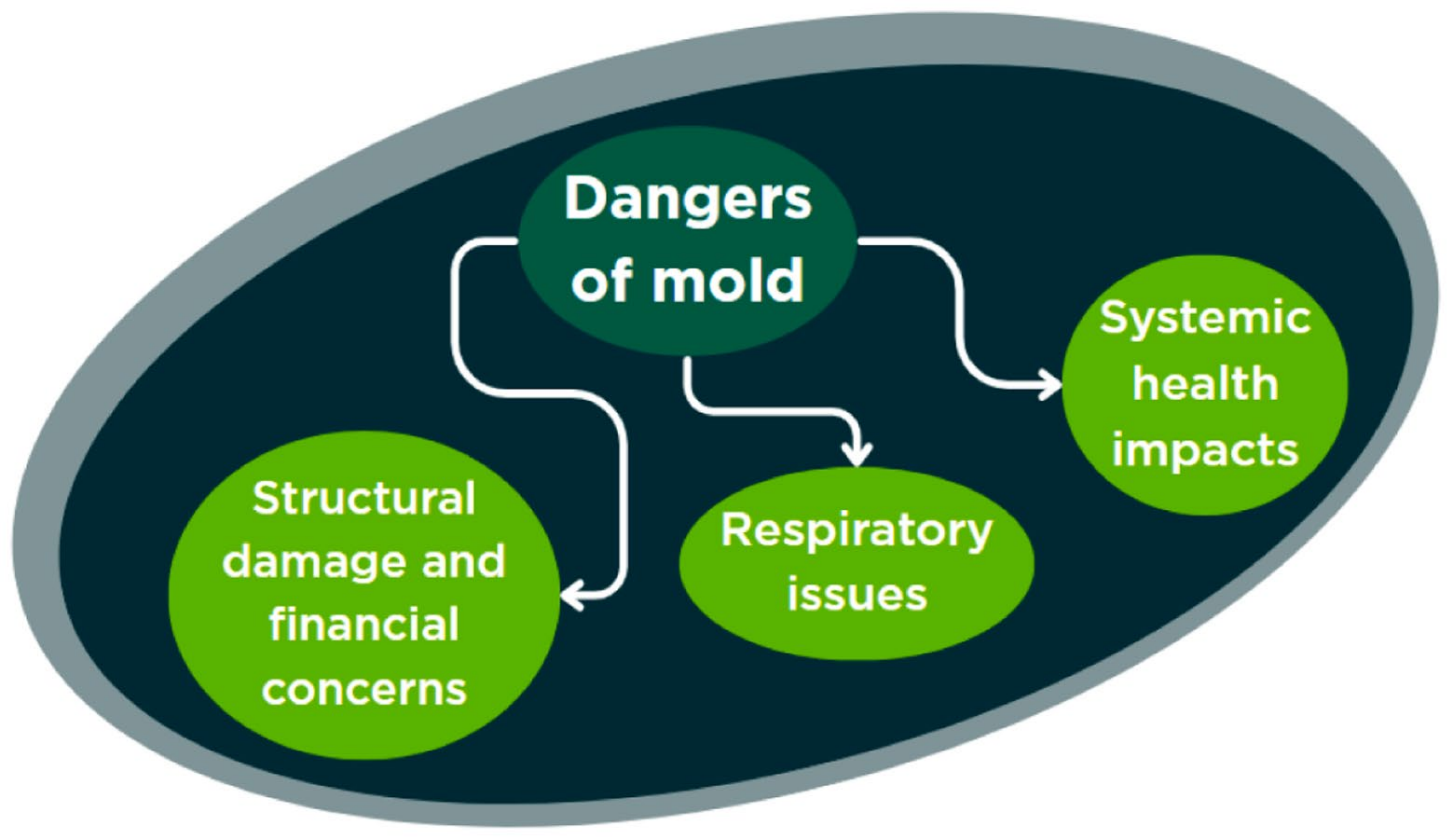
Themes of mold danger perceptions.

The data indicate that respiratory health concerns were the most prominent issue raised by all the participants in relation to mold exposure. Participants consistently associated mold with a range of respiratory problems, including allergies, infections, and broader immune-related effects. For instance, Participant 1 stated, “I always think of respiratory problems, but I also think of getting sick or easy—like immune disease caused by it” (P1), reflecting a perception that mold can contribute to both acute and chronic health outcomes. Several participants discussed the mechanisms by which mold may affect health, with Participant 6 expressing concern about inhalation: “My fear is respiratory types of things that can come from getting mold or the spores into your lungs” (P6). The potential severity of certain mold types was highlighted by Participant 7, who stated, “I’ve heard about black molds that can be really, really bad; like cause lung infections and stuff” (P7). Air quality was also a recurring theme, as Participant 10 observed, “Air quality, and because the spores kind of are throughout—can move throughout the air, getting them in your lungs” (P10). Figure 1 shows the results placed into one of the components of the HBM—danger of the problem and susceptibility to the threat.

#### 3.2.2 Systemic health impacts

The majority of participants in this study indicated serious health concerns that they perceived were attributed to mold exposure. Beyond the respiratory concerns highlighted previously, many participants voiced worries about the overall impact of mold on health and the immune system. Participant 1 expressed this fear by saying, “I always think the possibility of inhalation and mold, I think, could get in your bloodstream and cause sickness overall, just general really bad health” (P1). Another participant reflected on the range of symptoms: “Health concerns. Those with allergies it would make those worse. It can cause nose, skin, headaches, asthma, breathing troubles, cognitive troubles” (P19). This implies that mold exposure could weaken the immune system, making individuals more vulnerable to infections and allergic reactions. Participant 9 echoed, “It can also just make you physically ill in other ways. I mean you can, you know, it can upset your stomach or change, you know, your digestion if you get enough of it in your system, and it can cause other types of infections…” (P9). This comment highlights the potential for mold to cause gastrointestinal problems and other health issues, indicating a broader range of negative effects. Similarly, Participant 10 raised concerns about open wounds, mentioning, “Getting them in open sores and causing an infection” (P10). The range of concerns highlighted by participants indicated serious concern with both singular mold growth events (flood or major storm with no electricity) but also indicated concern over the long-term impacts of mold growth in their home environment.

#### 3.2.3 Structural damage and financial concerns

Participants also recognized the risk of mold to home structure, damaged home construction materials, and finances, which can lead to costly repairs and decreases in property value. Participant 2 expressed this concern, saying, “Yeah, certainly like deteriorating softer materials in your home. So, it could decompose or structurally impair certain things. So, resell value goes down, cost of—having to consistently repair certain areas” (P2). Likewise, Participant 13 said, “Well, of course, it’s going to grow in your wet wood and destroy your wood. So, it’s going to invade your wood and invite in termites and ants and everything else. It’s not a good thing to have in the house” (P13). These reinforce the idea that mold can significantly threaten a home’s physical structure. Participant 9 linked mold to underlying moisture issues, explaining, “Usually, you are not going to have mold unless you already have some source of moisture that you were not intending to have. It could be a roof leak, a leaky pipe and that could cause other structural damage in the long run… You know, the structural integrity of the house is going to decline the more you have kind of things kind of growing and infiltrating those structures.” (P9).

### 3.3 Participants’ actions to prevent mold impact

**Figure 2:**
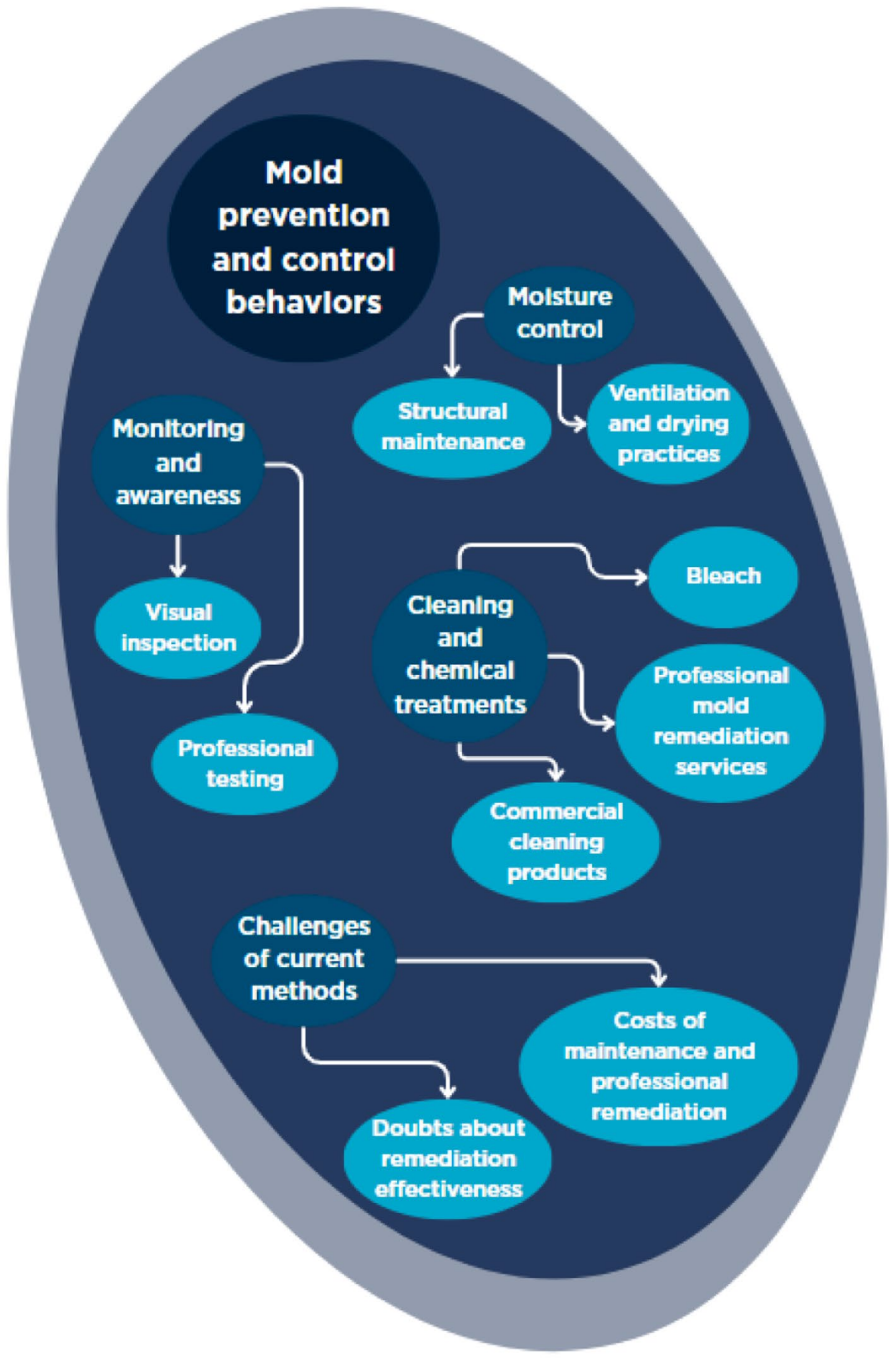
Themes of mold prevention and control behaviors.

Figure 2 summarizes the interview results with regard to another important component of the HBM actions and interventions.

#### 3.3.1 Monitoring and awareness

Participants demonstrated a high level of vigilance regarding mold risks in their homes, with regular monitoring and heightened awareness forming the foundation of their prevention strategies. Visual inspection was a routine practice, often prompted by past experiences or increased knowledge about mold. One participant reflected on this shift, stating, “I think, I mean, just maybe with age, I became more aware that I [as a homeowner] should be cleaning it as I see it” (P1). Another described a proactive approach to identifying problem areas: “I see mold too. And on the side of the house here, that’s probably the second most prevalent place” (P6). The presence of visible mold was often interpreted as an indicator of a more significant underlying issue, as one participant explained, “If—if it gets it to the point where I can see it, I know we have a problem.” (P9).

Awareness of health implications, particularly allergies, also influenced participants’ vigilance. For some, personal or family health concerns heightened their motivation to monitor and address mold. One participant shared, “I’m someone who has very obvious allergies. I have definitely known people who did not know they were very sensitive to mold and then have been in places and gotten, you know, sick from—from being in mold infested places” (P9). Another emphasized the importance of protecting family members: “As I said with my daughter-in-law, I certainly would not want to have anything around here that would affect her or the rest of the family or friends” (P8).

In addition to self-monitoring, some participants considered or utilized professional testing to confirm the presence or absence of mold, especially after incidents such as leaks or flooding. As one participant described, “I’m 90 percent convinced that I’m going to have someone come out and actually test for mold in like a year or so after the leak is fixed because I want to make sure that that leak did not end up causing mold” (P9). This approach reflects a desire for objective assessment beyond what is visible, particularly in situations where the risk of hidden mold is perceived to be high.

#### 3.3.2 Moisture control

Controlling moisture was the most consistently reported and emphasized strategy for preventing mold growth. Participants described a variety of methods aimed at reducing humidity and preventing water accumulation in their homes. Ventilation was a key theme, with many participants employing strategies to improve airflow and reduce dampness. For example, participants highlighted the importance of keeping doors or windows open to facilitate air circulation. One of them mentioned: “I try to keep all the doors to that back room open it feels like it gets a little bit more air through there, so it cycles better” (P2); Participant 12 emphasized, “I do open my windows for fresh air certain times of the year” (P12). The use of exhaust fans was also common, as one participant noted, “We do have ceiling vents in both bathrooms and I did put a vent above that right outside the door. So, we try to get the moisture out” (P4). Others described being deliberate about running exhaust fans and leaving doors open after showers to ensure thorough drying: “I try to be kind of conscientious in the master bathroom leaving the exhaust vent running for a while. Leave the door open” (P11).

Drying practices extended beyond ventilation. Frequent washing and drying of towels were another common practice: “I will for sure cycle my shower towels and bathroom towels a lot more. I feel like that’s just a potential for something damp, so I just try to wash them a lot” (P2). This participant also described leaving the washing machine door open after use and keeping the shower curtain partially open to prevent mold growth: “I leave the washer open the shower stays half open, half closed because I do not want that inside curtain to get mold” (P2).

Structural maintenance was also recognized as essential for moisture control. Several participants described addressing issues such as roof leaks or rotted wood to prevent water intrusion. For instance, one participant noted, “That was rotted wood around the chimney. So then we had to have a guy contractor come and replace some wood up there” (P8). Another emphasized the importance of roof repairs: “The first thing we did was re-did the roof” (P7). Concerns about older building materials and their ability to keep out moisture were also raised: “Well, again this whole side of the house you have got brick and it’s probably not doing a very good job of preventing moisture coming through.” (P3).

Regular maintenance of HVAC systems was another reported strategy, with participants aiming to change filters on a consistent schedule to maintain air quality and reduce moisture. One participant stated, “I try to do it [change HVAC filter] every month. And then I do not always do that, sometimes it’s two months” (P4). The use of dehumidifiers, though less common, was mentioned as an effective way to manage indoor humidity: “We run a dehumidifier a lot. It gets on my nerves, and then to empty it. And so we have a tube that we can run in his office. It goes through to the basement where there’s a dehumidifier running that’s pumped out” (P3).

#### 3.3.3 Cleaning and chemical treatments

Cleaning and remediation practices were central to participants’ efforts to manage mold, particularly when prevention measures were insufficient. Many participants relied on regular cleaning, often using bleach or other mold-specific products. The use of bleach solutions was the most frequently reported method of cleaning. For example, one participant explained, “I was born and raised in the south, so I use bleach. Yeah, I normally just make a bleach water mixture and just spritz that on, let it sit, wipe it down, rinse it with water” (P1).

Some participants also used commercial cleaning products, though there was skepticism about their effectiveness compared to bleach. One participant stated, “We scraped the paint off and then used a chemical treatment, which is the hydrogen peroxide and vinegar. I’m not sure about the validity of that, but it worked for us” (P16). Another emphasized, “Yeah, like Scrubbing Bubbles that does not have bleach. I do not trust that. It’s so good for cleaning, but it does not do the mold” (P1). Others mentioned using Lysol and other disinfectants, but often described these as providing only temporary relief: “I usually use like a disinfectant on it like Lysol it gets rid of the surficial expression of it at least temporarily” (P5). In some cases, participants experimented with different products based on research or recommendations, but the consensus was that commercial cleaners were less reliable for long-term mold control.

For more severe or persistent mold problems, professional remediation services were sought. Participants described situations where self-treatment was insufficient, leading them to contact specialized companies for comprehensive cleaning and, in some cases, mold testing: “Eventually, we had a company come in and clean that up. It was tested, and I cannot remember what the mold was” (P4). These accounts suggest that while most participants preferred to manage minor mold issues themselves, professional services were considered necessary for larger or recurring problems. However, many participants also mentioned challenges in interacting with those companies.

#### 3.3.4 Limitations and challenges

Despite their proactive efforts, participants faced several significant barriers to effective mold prevention and remediation. Cost was the most frequently cited, affecting both the ability to undertake major repairs and to access professional services. One participant described the challenge of finding affordable remediation: “especially with the crawlspace thing, trying to find a company that somebody recommends they have gotten estimates from $5,000 to $30,000” (P3). For some, limited budgets meant deferring necessary repairs: “Like, if I had an unlimited budget, I would redo the roof. We would probably like a professional estimate on, like, everything and anything we need to do to get to fix that we just aren’t doing that because we do not have a lot of savings and we are students” (P5).

Participants also expressed doubts about the effectiveness of many cleaning products available on the market. Comparisons between bleach and commercial products often favored bleach for its perceived efficacy: “the products at the store do not work as well as just the bleach and the half and half” (P1). Others acknowledged that while commercial disinfectants might provide temporary results, they did not address the root cause: “It does not really seem to do any long-term or it does not seem to like fix the problem. I’m sure the mold is like living deep inside of the wall.” (P5).

These limitations highlight the complexity of mold management in residential settings. Financial barriers and doubts about product effectiveness often constrained participants’ ability to implement more comprehensive or lasting solutions, despite their awareness and proactive attitudes. These accounts suggest that structural vulnerability (aging housing, recurrent flooding) and economic constraints (limited resources for professional remediation) interacted with reliance on visible cues to shape perceived susceptibility: respondents framed themselves as “at risk” mainly when mold was both visible and financially manageable to address, while invisible or chronic exposures were downplayed despite underlying concern.

### 3.4 Participant perspectives on genetically engineered microbiomes for mold remediation

**Figure 3:**
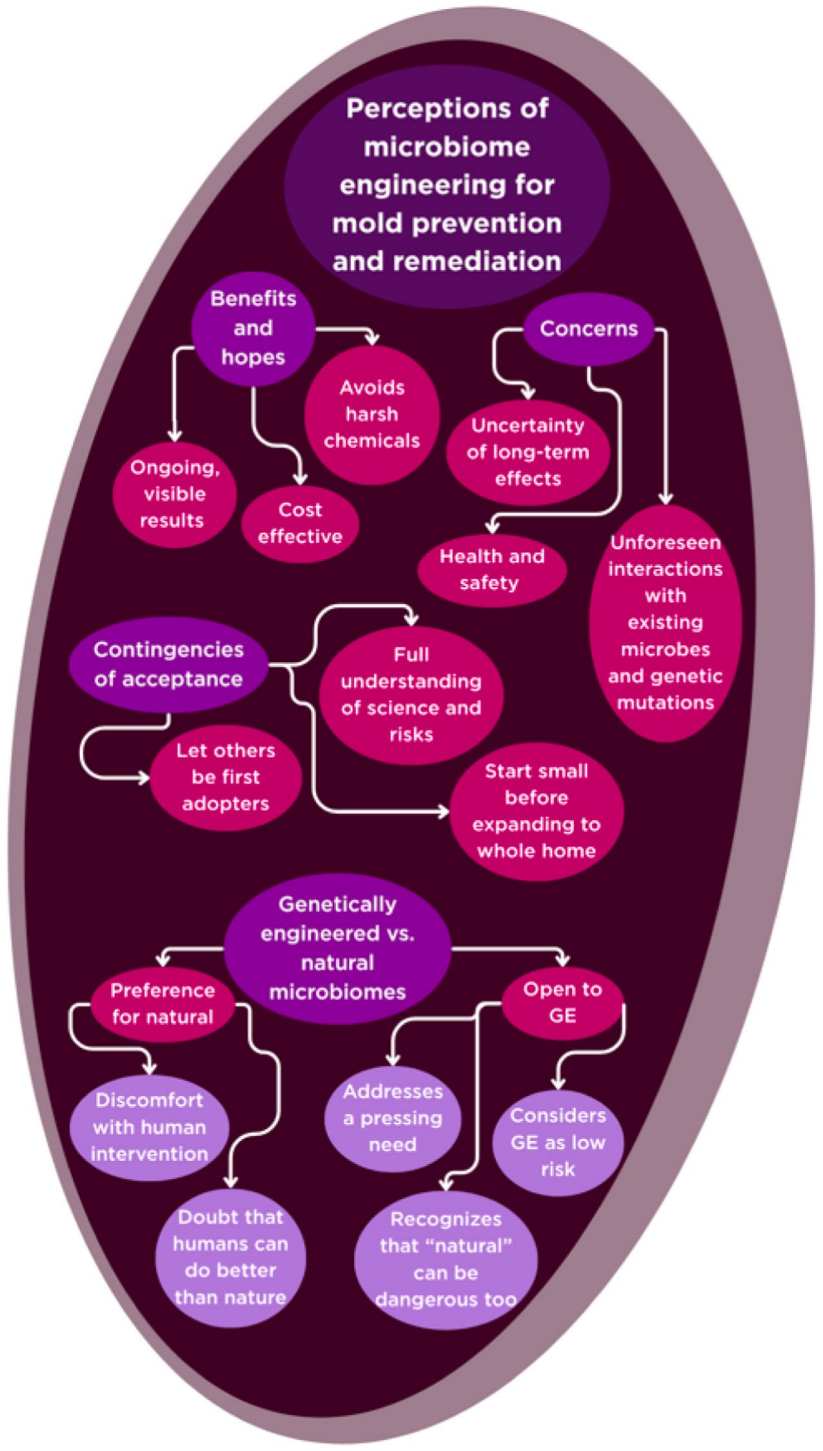
Themes of microbiome engineering perceptions.

Participants shared a nuanced and multifaceted view of genetically engineered (GE) microbiomes as a tool for mold remediation in the home. Their responses reflected a balance of hope, skepticism, curiosity, and caution, shaped by personal values, lived experiences, and varying levels of trust in science and technology. Figure 3 summarizes these results in the framing of the HBM’s benefits, hopes, and concerns of interventions.

#### 3.4.1 Benefits and hopes

Many participants saw clear potential in the use of GE microbiomes, especially when compared to traditional chemical-based mold treatments. The idea of a solution that could work continuously and naturally, without the need for harsh chemicals, was particularly appealing. As Participant 12 explained, “Just the X out of the harsh chemicals would be a huge benefit, that you do not have to—if they really engineered it to go in the HVAC system, you do not need to think about it. They could just do their thing and protect you, which is super helpful” (P12). Others highlighted the promise of ongoing, self-sustaining protection, envisioning a microbiome that “would reduce mold in a way that you would feel like it’s constantly doing the work instead of like a one-time fix and hope this is the solution to the thing. You have an ongoing fight against the mold, I guess. That would be appealing. You would feel reassured that it’s continuing to work” (P17).

Cost-effectiveness and accessibility were also seen as possible advantages, with some participants speculating that microbiome-based solutions could be more affordable than large, one-time interventions. Participant 12 noted, “So potentially it could be a benefit with cost, but it depends on what the marketing [is]… And these things usually reproduce like crazy” (P12). The potential for early detection and monitoring was another benefit, as Participant 13 shared: “I like the detection ability. The potential benefits of the monitoring and detection. I think that would be very worthwhile. And then people can make a decision what they want to do” (P13). Tangible, visible results, such as seeing mold disappear and stay away, were important for building trust in the technology. Participant 18 expressed, “If I could see something growing in the petri dish and I could see results, then I would trust what I’m seeing… Seeing results would mean to me seeing it go away and stay away” (P18).

Several participants recognized the broader social value of such solutions, especially for vulnerable populations who may lack the resources to address mold problems through conventional means. As Participant 19 observed, “I do see a fair amount of people who do have mold, and they just do not have the ability to do anything about it” (P19).

#### 3.4.2 Concerns and reservations

Despite these perceived benefits, participants voiced significant concerns about the unknowns and possible unintended consequences of introducing GE microbiomes into their homes. A recurring theme was uncertainty about long-term effects and the possibility of unforeseen interactions with existing microbial communities. Participant 1 reflected, “I’m always cautious of humans taking a little too much autonomy over our environment and trying to manipulate it. We’ve just seen time and time again that that’s not always a good answer, rarely actually a good answer” (P1), reflecting a general skepticism about the efficacy and safety of environmental manipulation. Another echoed, “Anytime you introduce something, man introduces his own idea of how this can work where nature does it its own way, it’s like, oh, a lot can happen. … I would be leery. But worth a try for sure. Yeah, depending on what microorganisms they are using, could that potentially have harmful effects on us? Or could that part—that introduced microbiome mess with the microbiome that’s already in existence or natural too?” (P12).

Others worried about the potential for genetic mutations, the emergence of new health risks, or the technology not being fully thought through before widespread adoption. Participant 13 voiced, “It’s the unintended consequences with so many things I see today. It’s not being thought all the way through… In all cases, we know that bacteria, molds, and other materials can mutate. So, what you test today and say this is good, well, who knows a year or two down the road, is there a genetic mutation that causes another problem?” (P13). Health and safety were central to these concerns, particularly for families with children or those with a history of health issues. Participant 14 described, “I always worry about toxins with kids, like what they are breathing, what they are exposed to and they are touching, how it affects their bodies… I would definitely look into all those things; I’d want to read about it… Usually everything has a downfall, so just kind of being aware of what that is and deciding if it’s worth what the benefits we think would be” (P14). The desire for transparency, scientific backing, and clear communication was strong, with many expressing reluctance to be “guinea pigs” for unproven interventions. Participant 15 emphasized, “There are so many things that have to be tested first. And then you find out after 20 years, oh, it causes this kind of thing” (P15).

Practical issues such as cost, accessibility, and the complexity of use also factored into their reservations. Participant 18, for example, raised concerns about affordability and practicality: “If it was more affordable—I mean you are going back to the cost and I’m saying if insurance covers it, yes, I would be happy to do it. But if I’m having to pay up front for something that might happen, I probably would not be that interested” (P18).

#### 3.4.3 Contingencies of acceptance

When it came to the prospect of adopting GE microbiomes for mold remediation, participants’ feelings ranged from strong reluctance to cautious openness. Many described themselves as unlikely to be early adopters, preferring to wait until the technology had been proven safe and effective by others. Participant 13 stated, “I’d be the last person to want to employ that… I’m not going to be the early adopter. I’m going to be one of the last after I know it’s stable and works” (P13). Participant 15 echoed, “I probably would never try it. I’m sure… I’d rather somebody else try it for 20 years before I did” (P15). Participant 19 similarly described themselves as someone who would “hold back… I’ll let everybody else tell me what you found, good or bad” (P19).

However, some participants expressed conditional openness, especially if the technology addressed a pressing need or if they could start small, such as using it in a single room before expanding to the whole house. Participant 16 suggested, “I’d be open to trying something like that, but just on a small scale I would say. If we want to just focus on the bathroom, then that would be a concentrated or a small-scale thing that would not affect the rest of the house… I think it’s worth a shot, and we have tried a bunch of things” (P16).

Others were more curious and willing to consider the technology, provided they had access to clear information and could understand how it worked. Participant 17 said, “I would be interested to use it. Of course, not blindly, just putting it in my home without reading about it or understanding the risks. Yeah, if I learn more about it, not just blindly, we would try it… If we understood what it was more, or if there was more information about what’s going on; like the mechanism by which it works, I would be the first one to raise my hand and use this technology” (P17).

#### 3.4.4 Preferences: genetically engineered vs. natural microbiomes

Participants’ preferences between GE and natural microbiomes were shaped by a mix of personal philosophy, health experiences, and trust in science. Several voiced a strong preference for natural solutions, expressing discomfort with human intervention. Participant 12 was emphatic: “Right now I’m leaning towards the natural. Yes. Oh, my goodness, yes. I do not like when we booger stuff, yeah. I would prefer naturally occurring microbes” (P12). Participant 15 echoed, “I would strongly prefer the naturally occurring microbes. No, just the unknown. Somebody is going to engineer something that they think they can do better than what’s already there, and that does not make sense to me” (P15). Participant 18 also favored natural solutions, drawing a parallel to their food choices: “I try not to eat food that’s genetically modified, so why would I want to bring it into my house? I mean, I’ve been more for organic; why would I want to bring that into my house?” (P18).

Others, however, were more open to GE microbiomes or saw little difference between the two. Participant 16 stated, “I do not think I have a preference there. I’m not too afraid of the genetic engineered microbiomes. … I think it’s probably okay” (P16). Participant 17 went further, challenging the idea that GMOs are inherently riskier: “No, that does not bother me at all. … I do not see GMOs as being inherently more harmful than natural organisms… To me I do not see any GMO… as having anything more dangerous. It’s just had genes added to help it express whatever” (P17).

Many other participants, like Participants 13 and 19, offered more balanced perspectives, recognizing that both natural and engineered options have potential risks and benefits. Participant 13 reflected, “Both have their own consequences… I’m not going to say oh, it’s natural, it’s better. … One out of 10,000 people are allergic to aloe, but we are throwing aloe in everything” (P13). Participant 19 similarly observed, “We have many organic things that are dangerous for us, so whether we create something that ends up being dangerous for us, I think is—I mean, I do not know the statistics on that, but I think that there’s a good chance that those are better. So, I do not think one versus the other is more scary” (P19).

## 4 Discussion

**Figure 4:**
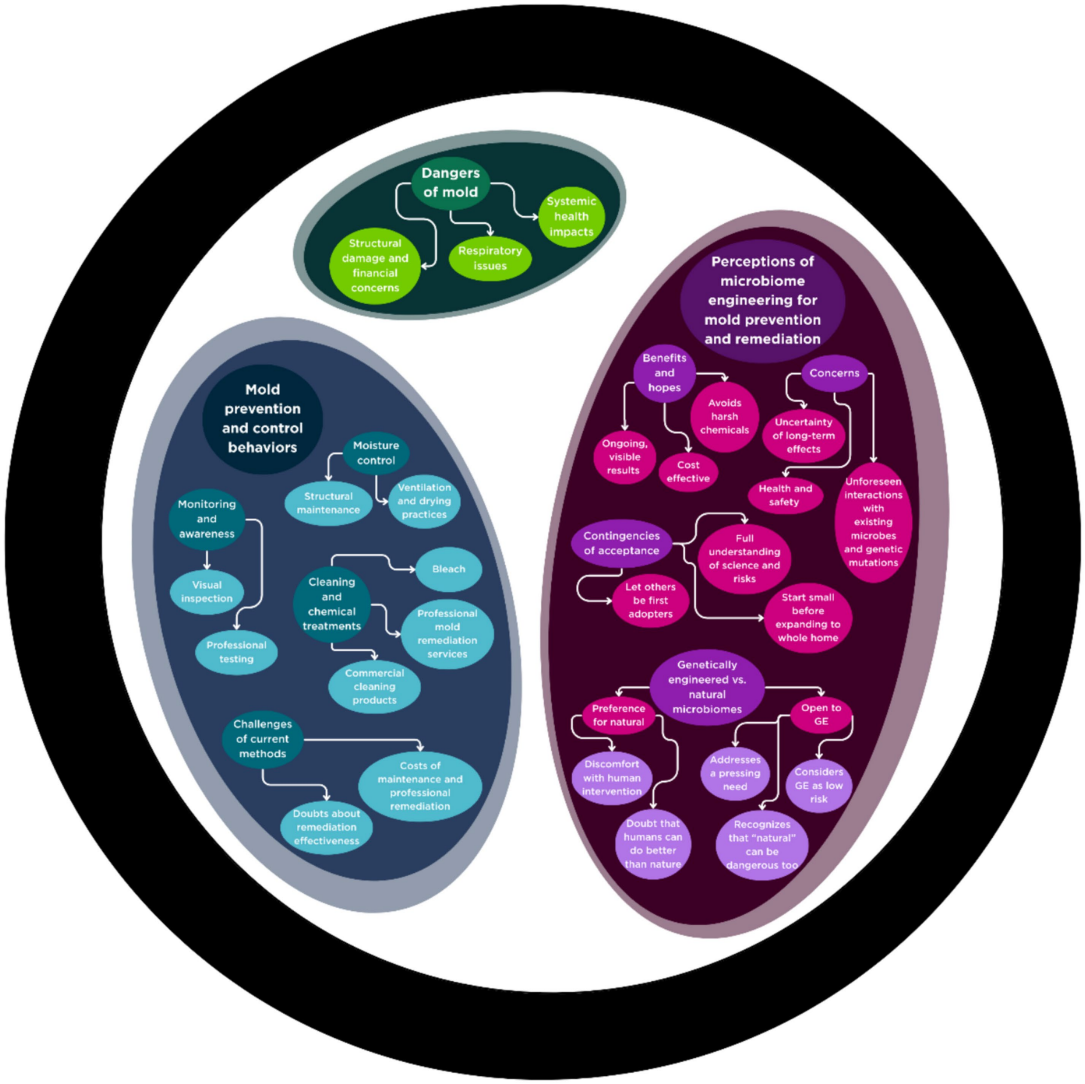
“Petri dish” visual of key themes about perceived dangers of mold, mold prevention and control behaviors, and perceptions of microbiome engineering for mold prevention and remediation.

This study explored how residents in Carteret County, NC, living in a coastal, high-humidity environment, understand and respond to mold in their homes, as well as how they perceive emerging microbiome-engineered technologies for remediation. Mold was not experienced as a single problem but as a multifaceted challenge shaped by health concerns, structural vulnerabilities, and financial limitations. Participants also viewed microbial interventions with a mixture of intrigue and skepticism, raising questions about safety, trust, and accessibility. The Health Belief Model (HBM), as summarized in Figures 1–3, provides a useful framework for interpreting these findings, as it highlights how perceptions of severity, susceptibility, benefits, and barriers structure responses to both everyday mold management and potential adoption of new technologies (Figure 4).

### 4.1 Mold as a biological and social reality

Residents described mold in many ways, sometimes simply as a fungus, sometimes as a dampness that breeds growth, and, most vividly, as something they could sense through smell and sight. These varied descriptions reflect what Aktas et al. (44) emphasize about mold: it carries multiple meanings depending on how people encounter it. Reliance on visible cues, such as stains, spots, and spreading colonies, meant that mold was often interpreted as a problem only once it became clearly observable, even though dangerous exposure can remain hidden. A few admitted they only recognized mold as a problem once it became visible, a behavior that echoes the warnings of Cox et al. (30) and Brambilla and Sangiorgio (14), who argue that this reliance on visible cues often leads people to underestimate the risks. From a Health Belief Model perspective, this pattern illustrates how perceived susceptibility is tightly coupled to what residents can see or smell, rather than to the less tangible, ongoing presence of airborne spores and hidden moisture.

Health concerns ran through almost every account. Participants spoke about respiratory problems such as coughing, wheezing, and allergies, and some also pointed to lingering or chronic effects. Their experiences line up with research by Dannemiller et al. (31), Brambilla and Sangiorgio (15), and others linking mold not only to asthma severity but also to long-term illness. But health was not the only worry. Many also described how mold damaged their homes, created anxieties about repairs, and threatened property value. This echoes patterns observed in Finland by Annila et al. (67), where mold was seen as both a health hazard and a financial burden tied to building integrity. For homeowners in Carteret County, the risks felt particularly weighty given the region’s high humidity and the regular battering of hurricanes. As Daniel et al. (50) note, in damp household environments, these environmental and socioeconomic pressures do not just add to the presence of mold, they make it an ongoing, difficult-to-manage challenge. Taken together, these accounts reveal that people perceived the severity of mold through both health and financial consequences. What is crucial is that this perceived severity was significantly amplified by structural vulnerabilities—things like precarious housing or limited resources—in ways that typical individual-focused models (that treat risk perception mainly as an individual cognitive appraisal, largely detached from structural conditions) often do not fully capture.

To reduce risk, residents described a range of strategies: improving ventilation, running dehumidifiers for long periods, maintaining roofs and HVAC systems, and, when possible, hiring professionals. Such measures are consistent with findings in China (51), France (48), and U. S. public housing (68), where household practices play a major role in shaping indoor microbial environments. Still, residents in Carteret County echoed what Daniel et al. (50) observed elsewhere: households do try to act, but costs, time, and systemic limitations often prevent them from dealing with deeper structural vulnerabilities. Within the Health Belief Model, this tension exposes a paradox. People recognized mold as serious, and they tried to act whenever they noticed warning signs like odors, stains, or allergy flare-ups. Yet economic barriers frequently stood in the way of consistent, effective action. In HBM terms, high perceived severity and salient cues to action (e.g., visible damage, symptoms) collided with substantial perceived barriers, especially financial constraints and limited control over building conditions, thereby constraining self-efficacy and limiting the translation of risk awareness into sustained preventive behavior.

In the end, mold in Carteret County was not seen solely as a biological hazard, it was also a lived and material struggle tied to the household, environment, and economy. This dual framing reflects trends seen internationally but is sharpened by the region’s geography and climate. As Brambilla and Sangiorgio (15) argue, tackling mold requires more than education or small behavioral changes. Lasting solutions must combine proactive, mold-aware building practices with support systems that help households protect their health in everyday life. Our findings thus extend HBM applications by illustrating how perceived susceptibility, severity, benefits, and barriers are co-produced by structural vulnerability and economic precarity, underscoring the need for interventions that address social and material conditions rather than focusing solely on individual behavior change.

### 4.2 Perceptions of microbiome-engineered solutions

Study participants were not only asked about everyday experiences with mold but also about emerging microbiome-based interventions being developed to address it. Their reactions revealed both curiosity and caution. Many welcomed the idea of using “good” microbes to control harmful mold, but they also raised some concerns about safety, environmental balance, and potential unintended side effects. This mix of interest and hesitation echoes national survey findings by Cummings et al. (39, 40), which show that US support for engineered microbiomes depends heavily on clear benefits, strong government oversight, and public trust in science. These responses align with broader sociotechnical scholarship showing that emerging biotechnologies are evaluated not only on technical merits, but through questions of control, fairness, and governance.

In Carteret County, this cautious outlook connects closely to what Hardwick et al. (57) describe as the sociotechnical realities of microbiome technologies. While engineers might frame these tools as straightforward technical fixes, residents interpreted them through a social lens: Were they fair? Could they be controlled? Would they align with everyday practices and ideas of cleanliness? Many preferred familiar, “natural” microbiomes. In Health Belief Model terms, people recognized potential benefits but saw significant barriers, uncertainty, invisibility, and concerns about altering natural systems, which limited enthusiasm. This emphasis on controllability and transparency echoes established patterns in the risk perception literature, in which invisible, expert-driven interventions tend to generate more concern and ambivalence than visible, user-controlled measures.

Even so, conditional openness was common. Residents said they might consider adopting new technologies if they could be proven safe and effective. This perspective reflects Singh and Rastogi's (59) argument that reframing mold management as an ecological practice could gradually shift public attitudes toward acceptance. Still, doubts remained strong, especially around risks like microbial imbalance or unintended gene transfer. Without trust and transparency, these fears could easily outweigh the promise of innovation. In HBM terms, trust in institutions and technology deployers and developers (implementers) functions as a critical modifier of perceived benefits and barriers: when trust is low, potential benefits are discounted and barriers loom larger, dampening willingness to adopt microbiome-engineering solutions even when perceived susceptibility and severity are high.

These views also temper the futuristic visions found in global “sociotechnical imaginaries” of microbiome engineering (40, 58). While concepts like self-sterilizing or regenerative buildings are often presented as ideal outcomes, Carteret County residents evaluated such ideas in light of lived realities: persistent dampness, the financial strain of upkeep, and the labor of constant mitigation. Their responses embody the “behavioral ambivalence” described by Cummings et al. (40), a willingness to explore new solutions, shadowed by deep skepticism and the fear of losing control to invisible processes. By situating microbiome-engineering proposals within everyday experiences of housing precarity and environmental risk, our findings show how local sociotechnical imaginaries can diverge from expert narratives, emphasizing accountability, reversibility, and the ability to opt out.

Equity was another core theme. As Hardwick et al. (57) and Singh and Rastogi (59) warn, without careful governance, microbiome interventions risk reinforcing inequities by being available only to wealthier households. Carteret County participants strongly illustrated this: families that struggle to afford bleach or HVAC servicing are unlikely to take on the costs of cutting-edge microbial tools. For such technologies to be genuinely acceptable, they will need to embody the principles of Responsible Research and Innovation (RRI), ensuring transparency, inclusivity, and sensitivity to cultural and economic contexts (39). Our study therefore contributes to sociotechnical and environmental health literatures by showing how equity concerns, trust, and perceived control intersect with HBM constructs to shape community receptivity to microbiome engineering, highlighting the importance of governance arrangements that are not only technically robust but socially attuned.

### 4.3 Limitations

This study offers valuable insights into how residents experience and respond to mold, yet it is important to acknowledge its limitations, of which one is the participant sample. Although we recruited homeowners from six different towns within Carteret County, NC, our study had more white participants with higher educational attainment and income. One reason is because we recruited homeowners only; renters were not recruited because they do not legally own the property that we needed to observe. Thus, our study demographics reflect homeowner demographics more than the general population in Carteret County. That is, homeowners are more likely to have a higher median income, more likely to have a college degree, be older in age, and identify as white. In addition, our sample was predominantly female, and thematic saturation was stronger among women than among men, which may limit the transferability of some findings to male homeowners.

Also, as with most qualitative work, our findings reflect the perspectives of a particular group of participants as well as the dynamics of the interview and observational settings. These dynamics were shaped by the social and professional positionality of the team (e.g., university-based researchers external to the community), which may have influenced participants’ comfort, disclosures, and emphases despite efforts at reflexivity, rapport-building, and attention to power dynamics. Moreover, the study was conducted in a single coastal county in North Carolina, characterized by specific environmental and socio-economic conditions (e.g., hurricane and flooding risk, local housing stock), which may limit the geographic transferability of our findings to other regions. We do not see these features as flaws, but as windows into the everyday uncertainties, constraints, and forms of knowledge that shape how people contend with mold in their lives. Future research should include renters and more demographically diverse samples across multiple geographic contexts, as well as how microbiome engineering technologies should be used by communities in practice. Our goal is not to claim universal truths but to highlight dynamics that can guide mold management approaches rooted in equity, trust, and sensitivity to community values.

## 5 Conclusion

This study highlights how mold in Carteret County, North Carolina, is far more than an environmental hazard. It is a persistent and deeply felt challenge that affects health, housing, finances, and the everyday lives of residents in this coastal community. For many, mold is not just an irritant but an embedded social and contextual issue, tied closely to broader economic, cultural, and environmental pressures.

The HBM helps explain these dynamics. Residents consistently recognized mold as a serious concern and often acted when prompted by clear cues such as odors or allergy symptoms. Still, their ability to respond was constrained by barriers like high costs, limited effectiveness of cleaning products, and structural housing problems. Because people relied heavily on what they could see or smell, hidden risks often slipped under the radar, reinforcing a cycle of reactive rather than preventive management.

When presented with the idea of microbiome-engineered tools for mold remediation, residents expressed cautious curiosity. While some valued the potential benefits, concerns about safety, trust, and control remained strong. The concept of using genetically engineered microbes—for instance, engineered mycoviruses to destroy pathogenic mold or genetically engineered microorganisms to displace mold—sparked particular questions about who would control these technologies and how their risks be managed. They wanted transparency, rigorous testing, and accountability before feeling comfortable with novel approaches. This cautious openness mirrors broader trends in responses to new biotechnologies: people are willing to consider them but only if development aligns with principles of fairness, accessibility, and cultural resonance.

Taken together, these findings suggest that successful mold management, whether through traditional measures or innovative technologies, cannot hinge on technical performance alone. Instead, solutions must be grounded in the lived realities of households, attentive to issues of trust and equity, and responsive to community knowledge and values. By integrating Responsible Research and Innovation (RRI) principles into the framing and development of microbiome tools, such as public inclusion, anticipation of risks, reflexivity of researchers and developers, and responsivity to public hopes and concerns, there is potential not only to address mold more effectively but also to contribute to healthier, more resilient living environments. Ultimately, lasting progress requires bridging scientific rigor with social meaning, ensuring that solutions are truly aligned with the people and places they are meant to serve.

## Data Availability

The datasets presented in this article are not readily available because the data are qualitative interview data of a small number of participants who share personal stories about their home and lives. Due to the sensitive nature of the data, there is very limited access to participants’ data. Requests to access the datasets should be directed to kdlandre@ncsu.edu.
