# Supplementary material for: Experiences with household mold and perceptions of microbiome engineering to mitigate mold
Source: Front Public Health. 2026 Jan 27;14:1725172. doi: 10.3389/fpubh.2026.1725172 (PMC12886478; doi:10.3389/fpubh.2026.1725172)
Supplement: Supplementary file 1 [file Table_1.DOCX]

**Supplemental Material**

**Semi-structured Interview Questions:**

Thank you so much for being here today. I’m a researcher with NC State University. We are talking to people about their understandings and experiences with mold. I appreciate the time you’ve taken to speak with us. You can stop the interview at any time – to ask a question, use the restroom, take a drink or water, or end the interview. Just let me know.

Before we begin, I want to note there’s no right or wrong answers here. It’s all about what you think and what you’ve experienced.

1. **Conceptualizations of mold:** First, we ask that you share your understanding of “mold.” Can you tell me what the word “mold” means to you?
   1. If participants seem hesitant or worried about getting the answer incorrect, encourage them that there’s no right or wrong answers. We are looking to understand what everyday people think.
   2. After the participant offers their definition and understanding of “mold”, you can offer the following script: “Thanks for sharing your understanding of mold. Before moving on with the interview, we want to offer a few thoughts on mold. We will consider mold as a type of fungal growth that forms and spreads in various kinds of damp conditions. Molds are sometimes referred to as mildew. Molds reproduce by releasing tiny spores that float through the air until landing in other places and potentially creating a new colony of mold. Molds can grow indoors and outdoors.”
2. **Dangers of mold:** What types of dangers does mold pose to you and your family?
   1. Suggestion if participants have trouble coming up with ideas: For example, are there dangers of mold to health, aesthetics of the home (the way your home looks), finances (like costs of cleaning up the mold)?
3. **Health impact perceptions of mold (if not addressed previously)**: How do you think mold in your home may impact your health?
   1. Suggestion if participants have trouble: Do you think mold in your home may impact your or your family’s skin or breathing, for example?
4. **General experiences with mold:** What are your experiences with mold in your home? For example, are there particular items or locations in your homes where mold has grown? What makes you think it’s mold–maybe by the way it looks or smells or something else?
   1. Suggestion if participants have trouble with experiences and examples: What about your air ducts, bathroom showers, countertops, refrigerator water dispensers, or anywhere else that’s commonly damp or wet?
5. **Observations of mold growth patterns:** Have you noticed patterns of mold growth or smells of mold and dampness in your home related to seasons? weather events, such as flooding? power outages? (probe for elaboration on examples)
6. **Cognitive and behavioral reactions to mold**: What do you think and do when you see mold growing in your home? Have you changed your behavior at all due to seeing mold growth in your home?
   1. Suggestion if participants have trouble: Do you try to clean the mold? Does it come back?
   2. Suggestion if participants have trouble: Do you open windows for fresh air if you see mold?
   3. Suggestion if participants have trouble: Do you change the filters in your HVAC system when you see mold or to prevent mold?
7. **Reflections on behaviors related to mold growth (if not addressed in prior question):** Do you think your own behaviors or habits may help or hurt mold growth in your homes? In other words, what are some things you may do to promote mold growth in your home? What are some things you may do to lessen mold growth in your home?
8. **Remediation awareness and concerns:** Are you aware of any resources available to you if you wanted to remediate or clean up the mold?
   1. Follow up: What concerns, if any, do you have about the costs of remediating and cleaning up the mold in your home?
9. **Perceptions of using microbiome engineering to remediate mold:**  Now I’m going to read you a paragraph about a potential way to clean up mold and/or prevent dangerous mold from contaminating the indoor environment, like your home.

**Script**: In recent years, a new area of study has emerged at the intersection of microbiology, architecture, and environmental science. The new area of study is called “microbiome engineering in the built environment.” The “built environment” includes indoor spaces that humans have created, like houses and office buildings. A “microbiome” is a tiny ecosystem made up of microbes, such as bacteria and viruses. This new field of study is exploring how microbiomes could be intentionally controlled, designed, and introduced into human-made structures like homes, office buildings, or hospitals to achieve specific benefits. For example, researchers may create mold-resistant construction materials using engineered microbiomes. Or they may create mold-detecting HVAC systems that then deploy beneficial microbes to combat the mold. However, as with any new science and technology issue, the introduction of microbiomes may have unintended negative consequences, which are being evaluated as well.

- 1. Do you have any questions about the paragraph that I just read?
  2. How do you feel about microbiome engineering to clean up mold or prevent mold in your home?
  3. Do you have any concerns? Or do you foresee any risks?
  4. What benefits do you see to microbiome engineering?
  5. Would you be willing to use a new microbiome engineering technology in your own home? Why or why not?
  6. Would it matter to you whether the microbes used are genetically engineered with modern biotechnology methods or mixtures of naturally occurring microbes?

1. **Community mold observations:** Have you noticed any mold growth in other buildings in your community? If so, where? And please describe your observations and any concerns.
2. **Ideas for addressing mold:** How do you think the community could or should address mold concerns?
3. **Desire for more knowledge:** Is there anything about mold that you want to know more about?
4. **Big picture:** We know that you are very busy and there’s a lot of things to manage in your life. In the big picture, or considering everything else, how does mold compare to your other priorities, concerns, or responsibilities?
5. **Anything else to share**: Is there anything else you want to share? Do you have any other concerns about mold in your home or in other buildings in your community?
6. **Probe for future research interest:** In the future, researchers may sample households for fungal mold in the area. Would you be interested in learning more about participation in a household fungal mold sampling project in the future?
   1. If not interested in sampling your home, do you know of any family, friends, or neighbors in Carteret County who may be interested in having their household sampled for fungal mold? If yes, would you be comfortable providing their name and contact information? We would reach out to them to discuss the project at a later date.

**Thanks for your participation in this interview. As we conclude the interview, here is a mold factsheet and remediation information.** Hand them the printed brochures ([one-pager](https://epi.dph.ncdhhs.gov/oee/docs/mold_factsheet_10132023-fin.pdf) from NCDHHS, [longer version](https://www.epa.gov/sites/default/files/2016-10/documents/moldguide12.pdf) from EPA, longer version from EPA [in Spanish](https://espanol.epa.gov/sites/default/files/2015-08/documents/moldguide_sp_1.pdf)) and the link to [NCDHSS mold webpage](https://epi.dph.ncdhhs.gov/oee/a_z/mold.html)
